# Multilayer films of exfoliated 2D oxide nanosheets by electrospray deposition

**DOI:** 10.1038/s41598-022-12768-3

**Published:** 2022-05-23

**Authors:** Moritz Nunnenkamp, Karin J. H. van den Nieuwenhuijzen, Johan E. ten Elshof

**Affiliations:** MESA+ Institute for Nanotechnology, University of Twente, P.O. Box 217, 7500 AE Enschede, The Netherlands

**Keywords:** Materials for devices, Nanoscale materials

## Abstract

The potential of the electrospray deposition technique as new method to make nanosheet-based multilayer films is evaluated. Densely packed nanosheet-based films with thicknesses of 1–20 nm with rms roughnesses of 2.1–2.4 nm were fabricated on samples of 1 cm^2^ size with a growth rate of 0.5 nm/min. Electrosprayed Ti_0.87_O_2_ nanosheet films were successfully used as oriented growth templates for 40 nm perovskite SrRuO_3_ thin films grown by pulsed laser deposition. The electrospray method provides a fast and easy alternative to the more commonly used Langmuir–Blodgett (LB) deposition method for nanosheet films.

## Introduction

Two-dimensional materials are a continuously expanding family of nanomaterials that have been drawing a lot of attention ever since the discovery of graphene^1^. Besides the well-known van der Waals materials like graphene, MoS_2_ and h-BN, there are also metal oxide-derived 2D materials such as Ti_0.87_O_2_, Ca_2_Nb_3_O_10_, δ-MnO_2_ and many others^2^. These 2D-materials have a sheet-like shape, hence their name nanosheets. Oxide nanosheets typically have lateral sizes ranging from hundreds of nanometers to tens of micrometers in two dimensions, and a thickness of less than 2–3 nm in the 3rd dimension. Oxide nanosheets have been employed successfully as heteroepitaxial growth templates for subsequent growth of preferentially oriented piezoelectric Pb(Zr,Ti)O_3_, ferromagnetic SrRuO_3_ and low power electronics VO_2_ thin films, to name a few^3–6^. Applications as redox-active electrodes in supercapacitors and batteries have also been reported^7,8^.

While the synthesis and colloidal stability of oxide nanosheet dispersions are well known^2,5^, the main current challenge concerns the development of easily applicable and scalable film deposition methods. The state of the art method to form nanosheet films on substrates from 2D oxide nanosheet monocrystals is by using the Langmuir–Blodgett (LB) method, where exfoliated 2D nanosheets that were gathered at a liquid–air interface are distributed onto a substrate in the form of a monolayer^9^. The results that can be achieved with this method are highly satisfying concerning surface coverage, film roughness and degree of control over film thickness allowing for implementation in various fields of technology^5^. However, the LB process is also time consuming, which limits its applicability and is the reason why this method is mostly used for research purposes. Not only that a single procedure takes up to several hours of laboratory work, but also the fact that only one nanosheet monolayer per deposition cycle is possible limits its industrial use. Moreover, although the LB method is scalable to large area coverage in theory, the actual transfer of a nanosheet monolayer from a liquid–air interface onto a substrate becomes increasingly sensitive to disturbances as the substrate dimensions increase. This is why several alternatives to the LB method have been proposed more in recent years, including spincoating^10^ and single droplet assembly^11^.

We explored the idea of electrospraying exfoliated nanosheet dispersions as another alternative new fabrication route for making multilayer films by generating nanosheet-covered substrates. The mass flux of nanosheets that can be deposited via spraying is much larger than with LB and the method is more easily scalable. Although the relatively random distribution of nanosheets by spraying will never result in the densely packed monolayers without any film roughness that can be achieved with the LB, spincoating or droplet assembly methods mentioned above, process optimization should be able to result in films with low roughness that completely cover a substrate.

Electrospray deposition or electrospraying concerns the deposition of small droplets onto a substrate using a strong electric field^12^. Essentially, precursor solutions based on evaporating solvents are ejected from a nozzle. A high applied voltage between the ejecting syringe and a conductive substrate causes the precursor solution to be electrostatically polarized and dragged towards the substrate following the electric field. The high electric field causes the liquid jet to be dispersed into droplets in the size range of micrometers, before their deposition on the substrate takes place. The process is dependent on two major families of factors. On the one hand, the precursor solution with its viscosity, surface tension and conductivity strongly determines the quality of the achieved spray cone^12^. On the other hand, externally controlled process parameters like electric field strength (applied voltage, nozzle to substrate distance), humidity and solution flow rate influence the experimental outcome^13^. It has been shown that electrospraying of sol–gel precursors allows the production of nanoparticles with a high throughput^14^, validating the assumption that a high nanosheet throughput should be possible as well. Moreover, the limited thickness of nanosheet films derived from LB deposition is not an issue in electrospray deposition. Electrospraying as a fast technique to form low-roughness multilayer nanosheet films is therefore a viable alternative for more application oriented purposes.

In this work we demonstrate the formation of electrosprayed nanosheet films and explore their potential as growth templates for heteroepitaxial perovskite films. We aim to show that the random spraying characteristics of electrosprayed nanosheet films do not affect their suitability for crystal orientation and preferentially oriented growth of subsequently deposited perovskite thin films.

## Results and discussion

The right phase and morphology of the parent compound H_1.07_Ti_1.73_O_4_ was confirmed by XRD and SEM analysis (see Fig. S1). The H_1.07_Ti_1.73_O_4_ phase was exfoliated into Ti_0.87_O_2_ nanosheets as described in the Methods section. In order to compare electrospraying of nanosheet films with LB deposited films it was necessary to achieve full substrate coverage using spray deposition. A homemade setup with humidity control was used for the electrospraying process. A liquid solution with a water/DMSO volume ratio of 1:1 was used as a spraying solution. Each experimental run was performed on two different substrates with sizes of 1 cm^2^ each. For AFM analysis and the following growth experiments, silicon (111) substrates were used. To enhance the contrast in the SEM analysis, Si(111)/Pt substrates were used. All substrates were covered with a monolayer of PDDA to increase the polarity and hydrophilicity and ensure the spreading of the liquid droplets after their arrival on the surface. Post-process annealing at 400 °C was used to remove the PDDA monolayer and organic residues from the surface.

The time needed to ensure that at least one full monolayer of nanosheets had been deposited over the entire substrate was determined experimentally. It was found that the time strongly depended on the droplet size in the electrospraying process. Droplets with diameters larger than a few hundreds µm, and especially droplets in the mm range naturally have a small surface to volume ratio compared to smaller droplets. This causes solvent evaporation in air and on the heated substrate to be insufficient for large droplets to evaporate entirely before the next droplets arrive at the surface. The resulting excess of liquid over time periods longer than tens of seconds caused the uniform film morphology to be disrupted upon impact, thereby washing off previously deposited nanosheets as a consequence. Smaller droplets with a higher surface to volume ratio containing just sufficient numbers of nanosheets are therefore desirable. Droplet sizes were experimentally determined by limiting the spraying time to a short time frame of just 30 s, in which only individual droplets were formed on the surface, rather than that they overlapped each other. The DMSO solvent from the solution left a contour of the as-deposited droplets on the PDDA coated layer on the substrate. The droplet size could be approximated from the surrounding DMSO layer using SEM. Since there is only one visible circular drying feature per droplet, a constant contact radius drying mode as described by Yu et al.^15^ was assumed.

**Figure 1:**
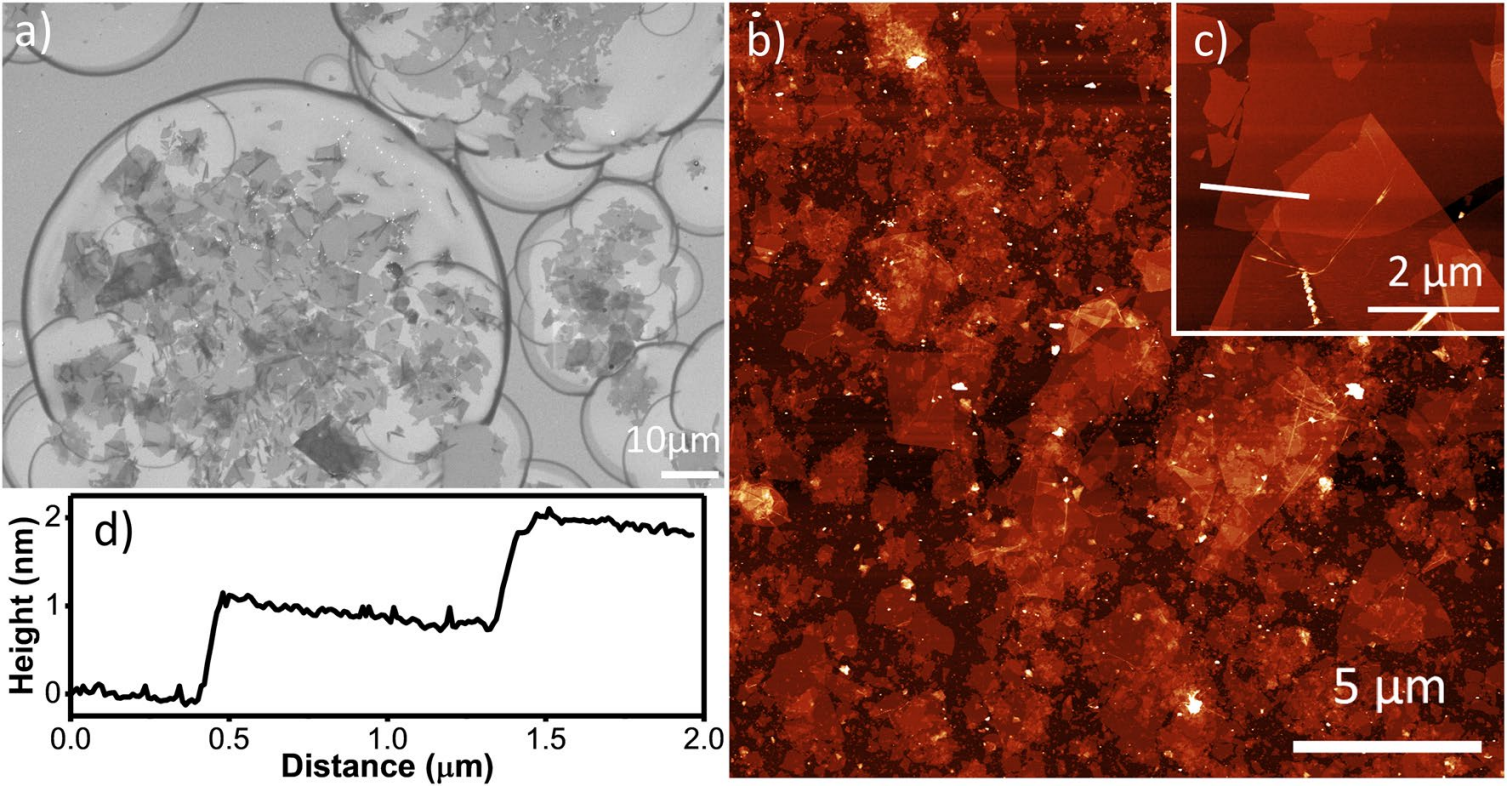
(**a**) SEM image of electrosprayed Ti_0.87_O_2_ nanosheets only partly covering the Si substrate before thermal annealing, (**b**, **c**) AFM image of electrosprayed Ti_0.87_O_2_ nanosheet film after thermal annealing, (**d**) line height profile extracted from data in image (**c**).

As shown in Fig. 1a, the droplet size distribution is relatively wide with droplet diameters varying from ~ 10 to ~ 100 µm. This is indicative of unstable spraying behavior, since perfect spraying conditions would result in monodisperse droplets. The instability may have been caused by small air flows during the spraying process or slight variations in the electric field strength. In addition to the large variation in droplet sizes, it can be observed that some of the smaller droplets only contained very few nanosheets. Taking into account that the nanosheets are several µm in size laterally, droplets smaller than a few tens of µm are too small to contain more than a few nanosheets. The minimum droplet size should therefore exceed several tens of µm in order to have a sufficient number of nanosheets per droplet, but also be smaller than a few hundred of µm in order to prevent droplet accumulation at the substrate as discussed above.

After thermal annealing of substrates that had been covered with nanosheets during spray deposition for 1 min, AFM analysis was performed to deduct the nanosheet film roughness and the thickness of individual nanosheets. Spots where droplets deposited multiple layers of nanosheets (typically 1–5 layers) were present, as well as uncovered areas. The topography of a partially covered substrate can be seen in Fig. 1b,c. The root mean square (rms) values of substrates with incomplete nanosheet coverage were 2.1 nm on average, with a total height ranging from 0 to 10.5 nm. As shown in Fig. 1d, fitting step functions at the measured height profiles extracted from single sheets as shown in Fig. 1c yielded a nanosheet thickness of ~ 1 nm, which is very similar to the values of 1.0–1.3 nm reported elsewhere^16^.

**Figure 2:**
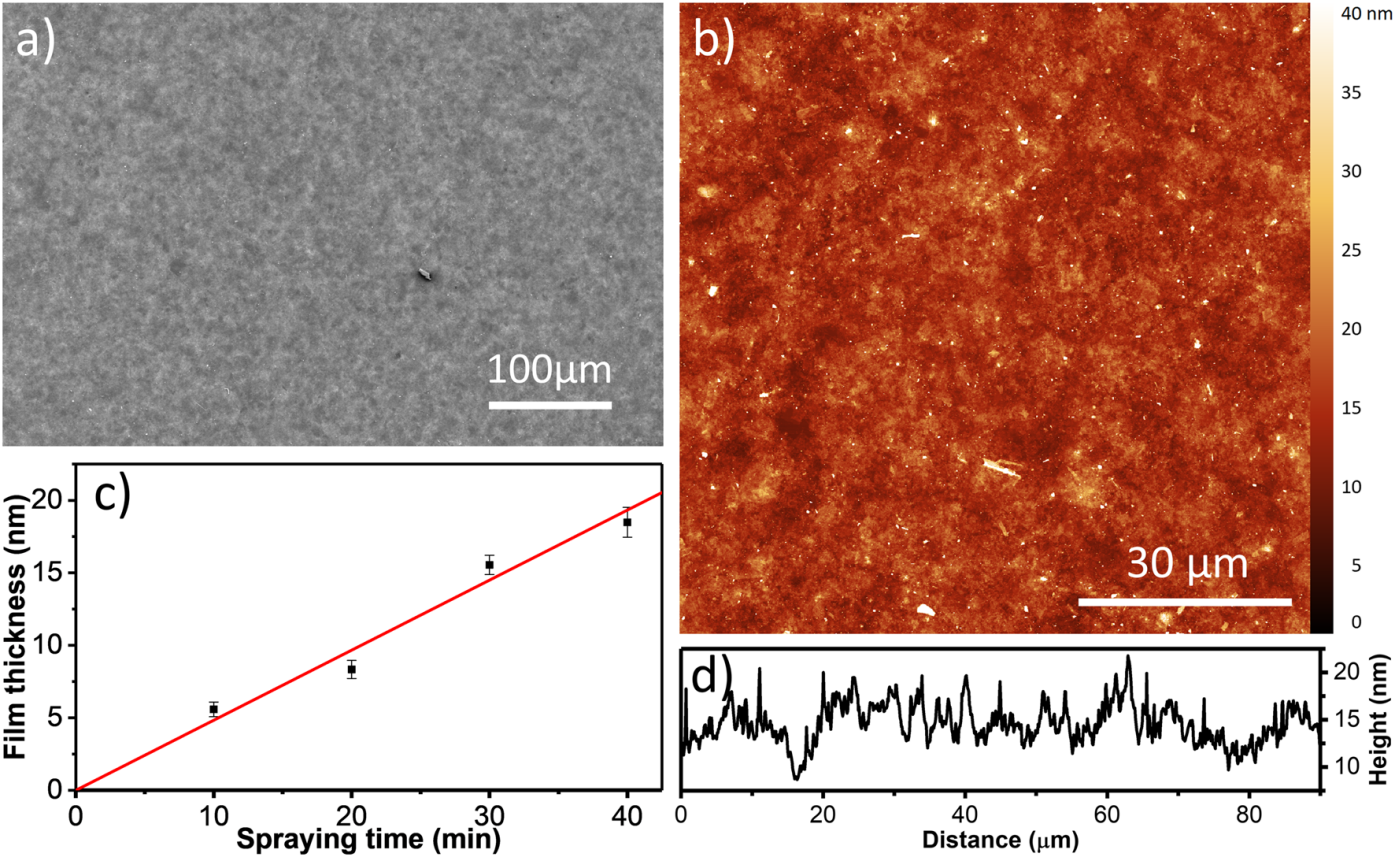
Electrosprayed nanosheets fully covering the substrate. (**a**) SEM image, (**b**) AFM image, (**c**) Film thickness versus spraying time, and the linear fit of the data points to determine the average deposition rate of the electrospraying process, (**d**) height line profile of film shown in (**c**).

Fully nanosheet-covered substrates employing varying spraying times were used to compare the morphologies of spray-dried films with LB-derived monolayer and *n*-layer substrates. Figure 2a shows that the electrosprayed film has a homogeneous thickness on large length scales, implying that the morphology derived from AFM analysis in Fig. 2b is representative for the entire surface. As shown by the line profile in Fig. 2d that was extracted from Fig. 2b, height variations of only a few nanometers over several tens of micrometers length are present in the investigated film. An rms value of 2.4 nm was calculated from the experimental data, which is very similar to the roughness of 2.1 nm of the partially covered substrate presented in Fig. 1a. Analysis of the topography on smaller length scales shows that the characteristic spray pattern where single droplets determined the local film thickness is also conserved for films with thicknesses in the range of several tens of nm. Similar to the image shown in Fig. 1a, local areas with higher film thickness appear to be present at locations where larger and/or multiple droplets had deposited nanosheets.

In order to gain further information on the initial spraying characteristics, fast Fourier transformation (FFT) of the presented data was done to convert the height profiles into frequency components. The FFT calculations did not show any dominant frequency, thus it can be concluded that no specific repetitive pattern is present in the sprayed films. It can be concluded that the droplets did indeed deposit in a fully random manner. The random spraying characteristic and the slow increase of rms roughness with film thickness implies that a film roughness not exceeding a few nanometer is expected even for thicker films.

The increased fabrication rate is one of the principal advantages of the electrospraying method as compared to LB deposition. Moreover, scale-up of the surface area to be coated is much less complicated than with LB. Partially masked substrates were used to determine film thicknesses via AFM step height measurements (see Fig. S2). The film thickness versus deposition time shown in Fig. 2c implies an average film growth rate of approximately 0.5 nm/min. This is significantly faster than LB deposition, which is limited to monolayer depositions where each layer typically takes 30–60 min to complete for a substrate of 10 × 10 mm^2^.

**Figure 3:**
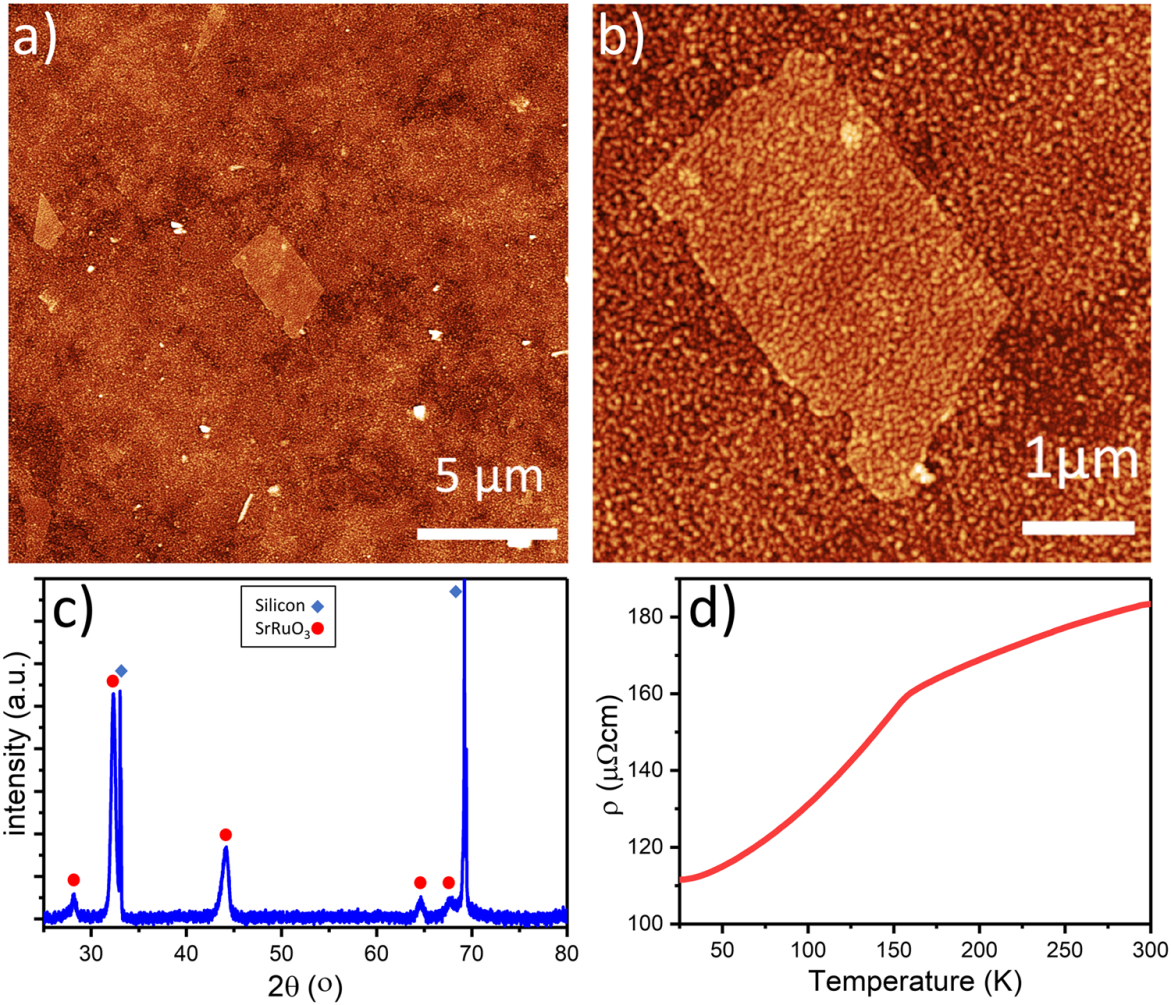
(**a**, **b**) AFM images of SrRuO_3_ film deposited on nanosheets at two different magnifications. (**c**) X-ray diffractograms of the deposited SrRuO_3_ film. The characteristic peaks of SrRuO_3_ and Si are indicated with red and blue dots, respectively. (**d**) Resistivity of the SrRuO_3_ film versus temperature.

We deposited SrRuO_3_ films by PLD on electrosprayed Ti_0.87_O_2_ nanosheet films to investigate their suitability as seed layers, and compared their film properties with those of SrRuO_3_ on LB films^17^. Figure 3a shows an AFM scan of a Ti_0.87_O_2_ nanosheet-based film fully covering a silicon substrate, and after subsequent growth of a 40 nm thick SrRuO_3_ layer on top of the nanosheets. The underlying nanosheet film was formed by using a spraying time of 20 min, which led to an average film thickness of 10 nm. The roughness of the SrRuO_3_ oxide film with an rms value of 2.3 nm was similar to that of the nanosheet seed film, much lower than the roughness of a PLD film directly deposited on Si (Fig. S3). Another relevant observation is that the morphological influence of larger single nanosheet is still clearly visible suggesting single crystal growth on individual nanosheets. As shown in Fig. 3b, the underlying nanosheet topography is preserved even after deposition step of 40 nm SrRuO_3_. Similar smooth film topographies that still show the morphology of the underlying seed layer are typical for layer-by-layer PLD growth of oxide films on oxide nanosheets^18^.

The XRD peak observed at 2θ = 32.3° in Fig. 3c matches well with the (110)_pc_ orientation of SrRuO_3_. Its high intensity compared to the relatively minor (002)_pc_ reflection of SrRuO_3_ at 2θ ~ 44° that is also present implies a high degree of (110)_pc_ oriented SrRuO_3_ film growth and a minority (001)_pc_ oriented fraction. These findings are in close agreement with data obtained on SrRuO_3_ films grown by PLD on LB-films of Ti_0.87_O_2_ nanosheets under similar conditions, as reported elsewhere^19^. The peak at 2θ = 67.7° can be attributed to the (022) orientation of SrRuO_3_^20^. Since SrRuO_3_ does not crystallize when grown directly on Si^19^, let alone with a preferential crystal orientation, these data indicate conclusively that the nanosheet seed layer facilitates both crystallization and (110)_pc_ film orientation, and that the degree of preferential growth orientation is similar as on LB-derived nanosheet films.

The resistivity of the SrRuO_3_ film shown in Fig. 3a,b versus temperature was measured using a physical property measurement system (PPMS). The obtained results are presented in Fig. 3d. A decrease in slope, thus resistivity increase after the temperature exceeded 150 K similar to the results published elsewhere^19,21^ is observed. The similarities in the resistivity versus temperature behavior again imply that there is no significant difference between functional perovskite films such as SrRuO_3_ when deposited on LB or electrosprayed nanosheet films as underlying growth template.

## Conclusions

A precursor solution suitable for electrospraying was made by exfoliation of H_1.07_Ti_1.73_O_4_ in a water/DMSO solution using TBAOH. Homogeneously distributed and fully substrate-covering multilayer Ti_0.87_O_2_ nanosheet films with thicknesses up to 20 nm were realized by electrospraying. The layer’s growth rate was t_l_ ≈ 0.5 nm/min. The electrosprayed films had similar properties as films formed by the LB deposition method. The RMS roughness values of 2.1–2.4 nm of (multilayer) electrospray films were found to be independent of the layer thickness. Crystalline SrRuO_3_ films grown by PLD on Ti_0.87_O_2_ nanosheets showed a preferential growth orientation and and had similar physical properties as similar films reported in literature that had been made using LB deposited Ti_0.87_O_2_ nanosheets as templates. The presented results show that electrospraying exfoliated nanosheets is indeed a feasible alternative to the LB deposition method, with the advantages of being simpler, less time consuming and more versatile in its application.

## Methods

The preparation of the layered parent compound K_0.8_Li_0.27_Ti_1.73_O_4_ and its protonation to form H_1.07_Ti_1.73_O_4_ was performed following the procedure by Sasaki and coworkers^22^.

### Exfoliation and solution preparation

H_1.07_Ti_1.73_O_4_ crystals (0.075 g) were dispersed in 15.0 ml of water and stirred for 30 min. 7.5 µl of Tetrabutylammoniumhydroxide (TBAOH) was added to the solution as an exfoliation agent and the solution was stirred for 3 h. The resulting solution containing Ti_0.87_O_2_ nanosheets was left to rest for another 30 min for the big entities to precipitate at the bottom of the solution. The top 10 ml of this stock solution were extracted and mixed with 10 ml of dimethyl sulfoxide (DMSO) leaving it stirring for another 1 h. The mixture was centrifuged for 10 min at 2000 rpm using a Thermo Fischer Biofuge Primo centrifuge to separate larger nanosheet agglomerates and other entities precipitate at the bottom of the flask. After centrifugation, the solution was left to rest for at least 24 h. The top 15 ml were extracted to be used for the electrospraying process.

### Electrospraying

Prior to electrospraying, the silicon substrate was treated with a 20 mmol aqueous poly-diallyldimethylammonium chloride (PDDA) solution. The substrates were soaked in the solution for 10 min to form a PDDA layer on top of the silicon substrate. The electrospraying process was performed on a homemade setup consisting of a spinneret grounded through a high voltage supply with applied voltages of 25 kV to a single metallic collector plate. The distance between the tip of the nozzle and the collector plate was kept constant at 12.5 cm. The inner diameter of the nozzle was 0.8 mm. The environmental factors were controlled in a homemade glovebox environment with which the humidity was controlled by slowly flushing the box with water vapor enriched dry nitrogen gas. The humidity was kept between 35 and 40% and the ambient temperature was kept at 22–24 °C. The substrate was heated to 50 °C. The flow rate was kept at 0.1 ml/h. The spraying time was varied between 30 s and 40 min, depending on the desired degree of substrate coverage. All measurement series were performed on PDDA coated silicon and silicon/platinum wafers of 1 cm^2^ in size. The coated substrates were then characterized using various methods. After spraying, the PDDA layer and other volatile residues were removed by annealing the substrate to 400 °C in air with a heating rate of 5 °C/min, followed by at least 30 min dwell time. Cooling down to room temperature was performed with a cooling rate of 5 °C/min.

### Pulsed laser deposition of SrRuO_3_

SrRuO_3_ films were deposited on Ti_0.87_O_2_ nanosheet films with pulsed laser deposition (PLD) using the conditions of Kuiper and co-workers^23^ in a 1:1 O_2_/Ar environment with a pressure of 0.30 mbar, with the heater operating at 670 °C. A square mask of 56 mm^2^ with rounded corners was used to select the most homogenous part of the laser beam, which was focused on the stoichiometric SrRuO_3_ target to a spot size of 1.8 mm^2^. The target was pre-ablated at 5 Hz for 6 min and deposition was performed at 1 Hz, using a fluence of 2.1 J cm^−2^ on the target. Depositions were carried out for 60 min, yielding a layer thickness of 40 nm as determined by AFM height analysis.

### Characterization

A Jeol JSM 6490 scanning electron microscope (SEM) used to analyze the morphology of the films was operated at voltages of 5–20 kV using magnifications varying from 100 to 2000 times. The accumulation voltage was varied between 3–5 kV, the spot size between 40–50 nm and the working distance was varied between 15–20 cm. The contrast and brightness were changed to achieve optimal contrast and visibility of measured features.

The Bruker Dimension Icon atomic force microscope (AFM) was used in the tapping mode in air to analyze the film topography. A silicon beam cantilever and tip provided by Budget Sensors was used with a nominal radius of 10 nm and a force constant of 40 N/m. The images were then processed and analyzed using Gwyddion software. The background was leveled using the polynomial background function using second order degree correction. Line artifacts were eliminated using the ‘align rows’ function.

X-ray diffraction (XRD) experiments were performed by high-resolution XRD on a Bruker D8 Discover with a Cu-Kα cathode in Bragg–Brentano geometry. The system was operated at 40 kV and 40 mA.

Samples were prepared in Van der Pauw geometry. Temperature-dependent Hall effect measurements were performed using a Quantum Design Physical Property Measurement System (PPMS by Quantum Design) in the temperature range of 10–300 K.

## Supplementary Information

Supplementary Information.
