# Supplementary material for: Multilayer films of exfoliated 2D oxide nanosheets by electrospray deposition
Source: Sci Rep. 2022 May 23;12:8673. doi: 10.1038/s41598-022-12768-3 (PMC9126931; doi:10.1038/s41598-022-12768-3)
Supplement: Supplementary file 1 — Supplementary Information. [file 41598_2022_12768_MOESM1_ESM.pdf]

SUPPORTING INFORMATION

Multilayer films of exfoliated 2D oxide nanosheets by electrospray deposition

Moritz Nunnenkamp, Karin J.H. van den Nieuwenhuijzen and Johan E. ten Elshof

MESA+ Institute for Nanotechnology, University of Twente, P.O. Box 217, 7500 AE Enschede, The Netherlands.

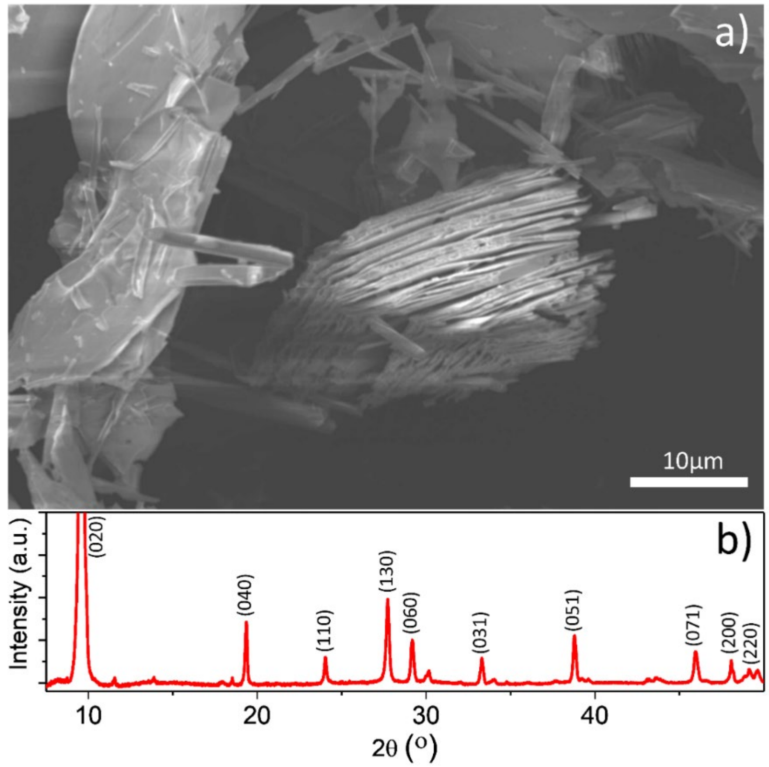

Figure S1: a) SEM image of $H_{1.07}Ti_{1.73}O_4$ powder showing the layered crystal structure; b) Powder XRD image of $H_{1.07}Ti_{1.73}O_4$ powder.

The parent compound has the desired composition and shape before the exfoliation process, as can be seen in Figure S1a, where the particle morphology as determined by SEM is suggestive of a layered crystal structure. Figure S1b confirms the crystal structure of $H_{1.07}Ti_{1.73}O_4$. The observed diffractogram is comparable to XRD data previously reported in literature[1].

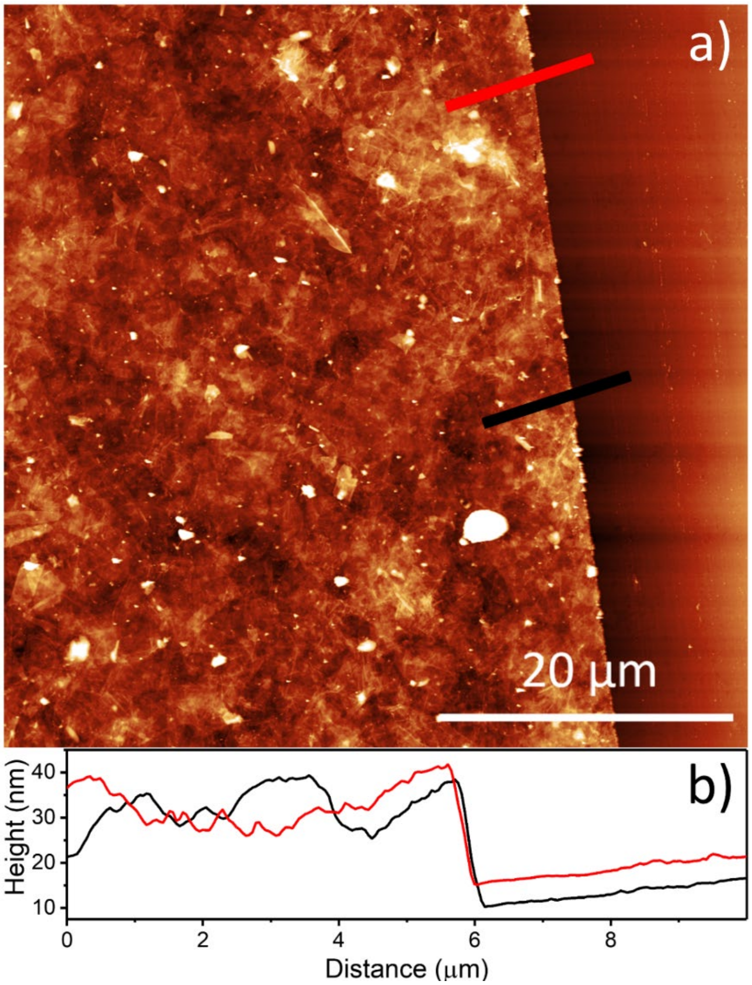

Figure S2: a) AFM image for step height measurement on fully nanosheet-covered substrates; b) two corresponding height profiles (red and black lines in Figure S2a) for determination of the deposited nanosheet layer thickness.

Figure S2 shows line profiles extracted from different areas of the AFM images. Each as-prepared sample was probed at four different locations and two line profiles per measured surface area were extracted. The line profiles were fitted with step functions to accurately find the individual step height. The average heights were used to determine the layer thicknesses and thus growth rate as shown in Figure 2c.

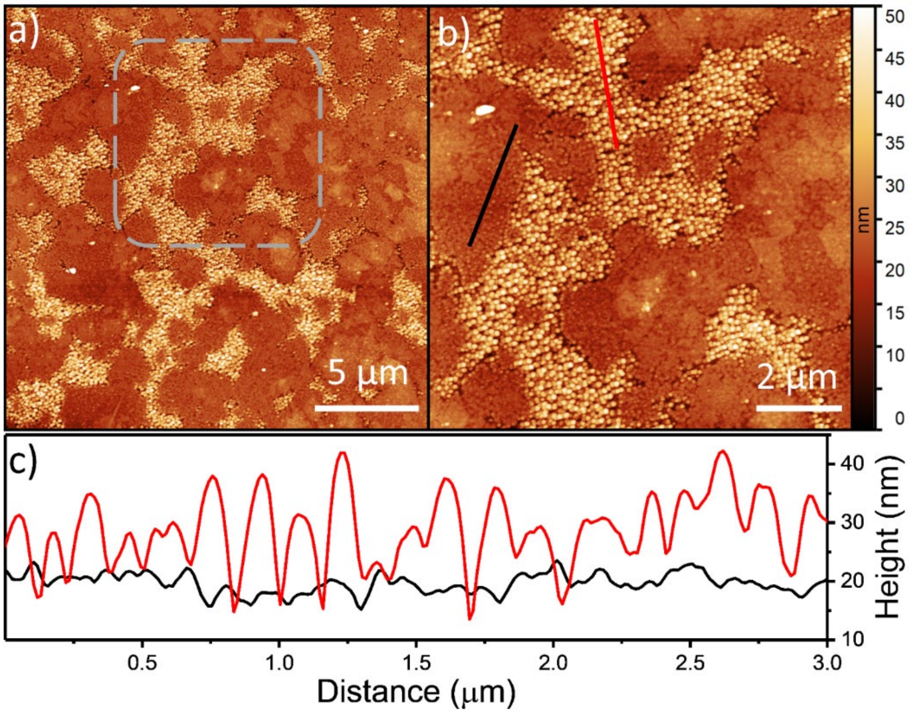

Figure S3: a,b) AFM data of $Ti_{0.87}O_2$ nanosheet film partly covering a silicon substrate with a PLD grown film of $SrRuO_3$ on top; c) Line profiles on $SrRuO_3$ films with silicon as a growth template (red line) and with $Ti_{0.87}O_2$ nanosheets as a growth template (black line).

Figure S3 a,b) show the AFM imaged topography of a $SrRuO_3$ film on top of a substrate only partly covered by $Ti_{0.87}O_2$ nanosheets. It is clearly visible that the crystal growth is enhanced by the usage of $Ti_{0.87}O_2$ nanosheets as a growth template. This can also be quantified by comparing the line profiles on either nanosheet templated growth (black) or bare silicon templated growth (red). The lower roughness of nanosheet-templated $SrRuO_3$ compared to the growth on bare silicon is a clear indication of improved film crystal quality.

Reference

[1]T. Sasaki, F. Kooli, M. Iida, Y. Michiue, S. Takenouchi, Y. Yajima, F. Izumi, B. C. Chakoumakos and M. Watanabe, Chemistry of materials **10** (12), 4123-4128 (1998).
